# Can plasma vitamin C predict survival in stage IV colorectal cancer patients? Results of a prospective cohort study

**DOI:** 10.3389/fnut.2023.1110405

**Published:** 2023-03-06

**Authors:** Sally Temraz, Jana Jabbour, Farah Nassar, Remie El Helou, Ruba Hadla, Maria Mezher, Ahmed El Lakkiss, Maya Charafeddine, Rihab Nasr, Ali Shamseddine

**Affiliations:** ^1^Department of Internal Medicine, American University of Beirut Medical Center, Beirut, Lebanon; ^2^Nutrition Program, Department of Natural Sciences, School of Arts and Sciences, Lebanese American University, Beirut, Lebanon; ^3^Department of Clinical Nutrition, American University of Beirut Medical Center, Beirut, Lebanon; ^4^Department of Anatomy, Cell Biology and Physiology, American University of Beirut Medical Center, Beirut, Lebanon

**Keywords:** colorectal cancer, plasma, progression, mortality, vitamin C

## Abstract

**Background and Aims:**

In light of the inconclusive evidence on the association between vitamin C status and colorectal cancer (CRC) outcome, this study assessed the prognostic value of vitamin C in participants with metastatic CRC (mCRC).

**Methods:**

Adults with mCRC and cancer-free controls were recruited in this prospective cohort study to allow for comparison of vitamin C levels with healthy individuals from the same population. Sociodemographic, lifestyle, medical variables, BRAF and KRAS mutations, as well as Vitamin C plasma level and food intake were evaluated. Predictors of diminished vitamin C level were assessed *via* multivariate logistic regression. Mortality and progression free survival (PFS) among mCRC participants were analyzed based on plasma vitamin C level.

**Results:**

The cancer group (*n* = 46) was older (mean age: 60 ± 14 vs. 42 ± 9.6, *p* = 0.047) and included more males (29% vs. 19%, *p* < 0.001) than the cancer-free group (*n* = 45). There was a non-significant difference in the vitamin C intake between the two groups; however, the mean plasma vitamin C level was lower in the cancer group (3.5 ± 3.7 vs. 9.2 ± 5.6 mg/l, *p* < 0.001). After adjusting for age and gender, the cancer group was more likely to be deficient compared to the cancer-free group [Adjusted Odds Ratio (95%CI): 5.4 (2.1–14)]. There was a non-significant trend for higher mortality in the vitamin C deficient cancer group (31% vs. 12%, *p* = 0.139). PFS did not differ based on vitamin C deficiency and patients with BRAF and KRAS mutations did not have significant differences in vitamin C levels.

**Conclusion:**

mCRC patients have lower plasma vitamin C levels than healthy controls. The trend toward higher mortality in the vitamin C deficient cancer group was not statistically significant. Whether this phenomenon affects survival and response to treatment warrants further exploration in phase III clinical trials.

## 1. Introduction

Colorectal cancer (CRC) is among the three most prevalent cancers in the world and in Lebanon (1–4). It has been estimated that 22% of diagnosed patients have metastatic CRC and 25% of those with localized tumor at presentation will develop advanced disease later on (5, 6). Vitamin C is a micronutrient that has been implicated in cancer metabolism and management. It has been speculated that vitamin C exerts an anti-cancer effect by pro-oxidant reactions and the formation of hydrogen peroxide and reactive oxygen species (ROS) which damage tumor cells leading to their death (7–11). The anticancer effect of vitamin C was also hypothesized to be mediated through the enhanced catalytic activity of TET enzymes which are responsible for the oxidation of 5-methylcytosine (5 mC), a well-known differentiation promoting agent (12). Furthermore, the antineoplastic role of vitamin C was demonstrated in CRC, specifically on KRAS and BRAF-mutated tumors. These mutations are prevalent in more than 50% of human CRC neoplasms and have been found to be more vulnerable to high dose vitamin C supplementation compared to other mutations (13).

However, data on the effect of vitamin C on survival in individuals with CRC are still conflicting. The majority of evidence shows limited effect of vitamin C supplementation on overall mortality among patients having CRC. Nevertheless, one study reported an association between vitamin C supplementation and decreased risk of CRC mortality before the age of 65 years and rectal cancer mortality at any age (14–17). Despite the scientific interest in the subject of vitamin C and CRC, there is inconclusive evidence on the association between vitamin C level, progression, and survival among individuals with CRC and those with KRAS and BRAF mutations. To address this gap, we assessed the prognostic and predictive value of vitamin C in patients with stage IV CRC by comparing vitamin C level between cancer-free and cancer patients and analyzing the specific plasma vitamin C levels in RAS (NRAS/KRAS) and BRAF mutated CRC patients.

## 2. Materials and methods

### 2.1. Study design and ethical considerations

This is a prospective cohort study conducted at the American University of Beirut Medical Center (AUBMC) from May 2018 until October 2020. The study cohort consisted of patients with metastatic CRC (mCRC), (*n* = 46) taken from a larger cohort whose primary aim is to assess the prognostic value of inflammatory markers and microRNA. The cancer cohort was compared to a group of “cancer-free” participants (*N* = 45). Newly diagnosed, treatment naïve, adults with stage IV CRC, presenting for treatment at AUBMC were considered eligible to be in the former cohort. Cancer-free adults (≥18 years) accompanying patients to the oncology unit at AUBMC as well as AUBMC employees were approached to join the latter group. Data related to the patients’ cancer diagnosis (date and stage at diagnosis, tumor location, imaging studies, laboratory studies, pathology reports, treatment given, etc.) and to the participants’ sociodemographic variables (date of birth, gender, weight, height, family history, past medical history, past surgical history, residential area, household income, number of people in household, number of rooms in the house) were collected from the clinical chart and the hospital admission charts. KRAS, NRAS, and BRAF mutations were assessed using the Idylla™ KRAS and NRAS-BRAF Mutation Tests. These *in vitro* diagnostic tests are performed on the Biocartis Idylla™ system. They allow the qualitative detection of 21 mutations in codons 12, 13, 59, 61, 117, and 146 of the KRAS gene, 18 mutations in codons 12, 13, 59, 61, 117, 146 of the NRAS gene, and 5 mutations in codon 600 of the BRAF gene. 5–10 μm sections of formalin-fixed paraffin-embedded (FFPE) human tissue from metastatic colorectal tumors are used in order to extract DNA for subsequent real-time polymerase chain reaction (RT-qPCR) amplification and detection. In comparison to a reference method, the Idylla™ mutation test demonstrated 96.7% overall diagnostic agreement for the KRAS mutation and 100 and 99.6% for the NRAS and BRAF genes, respectively (18). Progression Free Survival (PFS) was defined as the overall time participants with cancer lived without disease progression.

The study followed the ethical principles of the Declaration of Helsinki. Approval for the research protocol was obtained from the Institutional Review Board at the American University of Beirut prior to study initiation. Individuals who agreed to enroll in the study were informed about the benefits and risks of joining the study and signed a consent form prior to any study-related activity.

### 2.2. Vitamin C plasma level measurement

To assess vitamin C level, a venous blood sample (2 ml) was collected in Lithium-heparin tubes and was processed in the dark within 30 min of collection. The blood sample was centrifuged at 3500 rpm for 10 min to isolate plasma; 200ul of plasma was added to 200ul of a precipitation reagent [Immuchrom GmbH Vitamin C kit (Heppenheim, Germany)]. After vortexing, samples were left for 10 min at 2-8°C, and then centrifuged at 10000 × *g* for 10 min. The supernatant was then stored at –20°C for up to 1 month and 20 μl of the supernatant was later injected into the HPLC-system. Each sample was done in triplicate and run with a plasma calibrator provided by the kit. The chromatograms were recorded by a UV-detector. The quantification was performed by the delivered plasma calibrator; the concentration was calculated *via* integration of the peak areas. A cut off point of 5 mg/l was used to define vitamin C deficiency (19).

### 2.3. Diet and physical activity level

Vitamin C intake was assessed using a food frequency questionnaire (FFQ) for the Lebanese diet, adapted from a validated questionnaire (20). The tool inquired about the frequency and quantity of consumption of commonly consumed items in the Lebanese diet over the last 3 months. Two licensed dietitians, who received training and cross training, were responsible to perform the dietary assessment and to enter the collected data for analysis. The FFQ backend was developed using references from the FoodData central by the United States Department of Agriculture and local food composition tables (21, 22). It allowed for the assessment of the subject’s macro and micro nutrients intake. Vitamin C nutrient density was assessed by dividing intake over daily caloric intake. Activity level was assessed using the International Physical Activity Questionnaire (IPAQ)-short form that was validated against accelerometers and adapted to the Arabic language (23, 24). The IPAQ-SF looks into an individuals’ physical activity for the past 7 days as well as their walking and sitting times and categorizes individuals as having low, moderate, or high physical activity level.

### 2.4. Statistical analysis

Numerical and categorical variables were presented as mean and standard deviations and counts and relative frequencies, respectively. Categorical and numerical variables were compared between the two cohorts using the Chi square and student *t-*test, respectively, with significance considered for *p-*values <0.05. Progressive disease was plotted using the Kaplan Meier curve to calculate median time-to-progression, defined as the time from initial diagnosis to disease progression date or the end of follow-up (censored observations for subjects who did not reach the progression event). The backward conditional multivariate regression analysis was run to identify factors affecting vitamin C plasma levels. The following variables were assessed at the univariate level: Age, gender, Body Mass Index (BMI), smoking status, cancer diagnosis (control vs. case), and physical activity level. Variables with a *p-*value <0.10 at the univariate level were considered significant and incorporated in the multivariate model. A value of *p* < 0.05 was considered significant for the rest of the analyses. All statistical analyses were performed using the SPSS v.25.0 statistical package (IBM, Armonk, New York, United States).

## 3. Results

Metastatic CRC patients (*n* = 46) and cancer-free controls (*n* = 45) were included in this study and their descriptive data are presented in Table 1. Mean age and male gender were higher in the cancer group. Variables such as crowding index, smoking status, and mean BMI did not differ significantly between the two groups. Although there was no significant difference in vitamin C absolute dietary intake between the two groups, mean plasma vitamin C levels were almost double that of the cancer-free group (9.3 ± 5.5 vs. 4.2 ± 4.3, *p* < 0.001) (Table 1). Specifically, the mean plasma vitamin C level was 4.3 ± 4.3 for the colon group and 4.0 ± 4.2 for the rectum group. The mean difference in plasma vitamin C level between the control and colon groups was 5.07 (*p* < 0.0001). As for the mean difference in plasma vitamin C level between the control and rectum groups was 5.28 (*p* = 0.010). As for the mean difference between the colon and rectum groups it was 0.215 (*p* = 0.916) which was non-significant. The majority of the cancer cohort (63%) had vitamin C deficiency compared to 22% of the cancer-free group (*p* < 0.001).

**Table 1:** Descriptive data of cancer-free and colorectal cancer group.

Cancer			
Status variable	Cancer-free (*n* = 45)	Cancer (*n* = 46)	*p*-Value
Males, *n* (%)	19 (42)	29 (63)	**0.047**
Age (years), mean ± SD	42.3 ± 9.6	59.7 ± 13.7	**<0.001**
Crowding Index, mean ± SD	0.99 ± 0.33	0.92 ± 0.45	0.462
Smokers, *n* (%)	17 (38)	21 (54)	0.14
Mean BMI (Kg/m^2^), mean ± SD	26.3 ± 3.8	28.2 ± 5.6	0.061
Plasma vitamin C (mg/L), mean ± SD	9.3 ± 5.5	4.2 ± 4.3	**<0.001**
Deficient plasma vitamin C, *n* (%)	10 (22)	29 (63)	**<0.001**
Vitamin C intake (mg/day), mean ± SD	144 ± 83	172 ± 90	0.129
Nutrient density of vitamin C (mg/1000 Calories/day), mean ± SD	51.8 ± 21	59.3 ± 35	0.241
Comorbidities, *n* (%):			**<0.001**
None	38 (84.4%)	15 (32.6%)	
Hypertension	3 (6.7%)	11 (23.9%)	
Cardiac comorbidities	3 (6.7%)	14 (30.4%)	
Diabetes mellitus	1 (2.2%)	5 (10.9%)	
Renal comorbidities	0 (0%)	1 (2.2%)	

BMI, Body mass index; significant values are bolded.

The association of deficient vitamin C plasma level with several variables was investigated using univariate and multivariate logistic regression (Table 2). Age, gender, and presence of mCRC were incorporated in the multivariate logistic regression as these variables had *p* values of <0.10 at the univariate level. The only variable that was significant at the multivariate level, after adjusting for age and gender, was the presence of cancer. Participants having cancer had an adjusted odd ratio of 5.4 (95% Confidence Interval: 2.1–14) of having a deficient plasma vitamin C level (Table 2).

**Table 2:** Univariate and multivariate Logistic regression of deficient vitamin C plasma level.

Variable	Univariate analysis		Multivariate analysis	
	OR (95% CI)	*p-*Value	AOR (95% CI)	*p-*Value
Age (years)	0.97 (0.94–1.0)	0.062	0.98 (0.94–1.0)	0.374
Male	0.37 (0.155–0.870)	0.023	0.45 (0.18–1.2)	0.099
Body Mass Index (Kg/m^2^)	1.0 (0.96–1.1)	0.360		
Physical activity level				
Low (referent)	1	0.203		
Moderate	0.78 (0.25–2.5)	0.666		
High	0.15 (0.017–1.2)	0.146		
Smokers	1.0 (0.44–2.5)	0.933		
Daily Vitamin C intake (mg) per 1,000 Kcal	0.99 (0.97–1.0)	0.174		
Having colorectal cancer	6.0 (2.4–15)	<0.001	5.4 (2.1–14)	<0.001

Variables with *p-*values < 0.10 at the univariate level were entered in the model at the multivariate level. AOR, adjusted odds ratio; OR, odds ratio.

In the cancer group, there were 39 participants with colon cancer and 7 participants with rectal cancer. BRAF mutations occurred in 2 patients while RAS mutations (NRAS/KRAS) occurred in 24 patients. No significant differences in age, gender, cancer type, physical activity level, BMI, nutritional status at baseline and weight loss at 12 months were noted upon dividing CRC patients according to plasma vitamin C adequacy level (Table 3). Even though BRAF, KRAS and NRAS mutations, cancer progression and mortality were more prevalent in the vitamin C deficient group, the differences between groups did not reach statistical significance (12% in the adequate plasma vitamin C group vs. 31% in the low plasma vitamin C, *p* = 0.139) (Table 3).

**Table 3:** Colorectal cancer cohort characteristics in adequate and deficient plasma Vitamin C level.

Variable		Adequate plasma vitamin C (*n* = 17)	Deficient plasma vitamin C (*n* = 29)	*p-*Value
Age (years), mean ± SD		60.8 ± 14	59.1 ± 14	0.679
Gender, *n* (%)	Male	9 (53)	20 (69)	0.277
Cancer type	Colon	15 (88)	24 (83)	0.618
	Rectal	2 (12)	5 (17)	
Physical activity level at baseline, *n* (%)	Low	13 (76)	21 (75)	0.444
	Moderate	2 (12)	6 (21)	
	High	2 (12)	1 (4)	
BMI at baseline (Kg/m^2^), mean ± SD		28.8 ± 6.6	27.9 ± 5.1	0.591
Nutritional status at baseline, *n* (%)	Well nourished	10 (59)	11 (38)	0.386
	Moderately malnourished	6 (35)	15 (52)	
	Severely malnourished	1 (6)	3 (10)	
Mutations	BRAF	0 (0)	2 (6.9)	0.379
	KRAS	6 (35)	14 (48)	0.654
	NRAS	0 (0)	2 (6.9)	0.468
Disease status, *n* (%)	Partial response	2 (12)	2 (7)	0.431
	Complete response	1 (6)	7 (24)	
	Stable disease	2 (12)	2 (7)	
	Progression	12 (71)	18 (62)	
Weight loss at 12 months (Kg), mean ± SD		0.05 ± 0.2	−0.01 ± 0.2	0.363
Death, *n* (%)		2 (12)	9 (31)	0.139

**Figure 1:**
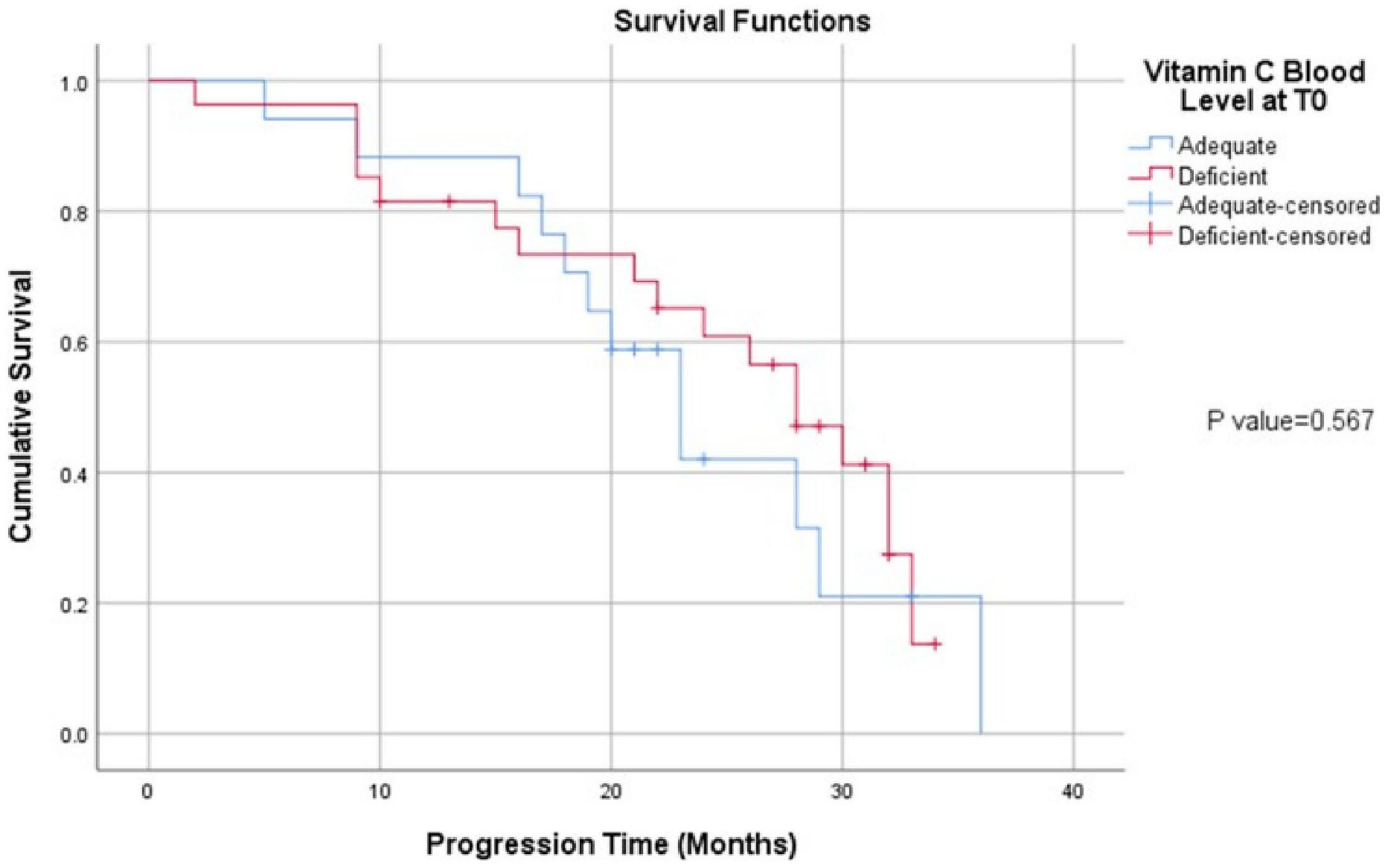
Kaplan Meier curve of progression free survival in CRC cohort divided according to plasma vitamin C levels.

At median follow up of 24 months, there were 22 patients (48%) who were still alive. Data on disease status was available for all patients. In total, there were 30 patients (65%) with disease progression. The overall median PFS for the cancer group was 28.0 months (95% CI: 22.8–33.2). The mean plasma vitamin C in the deficient group was 1.406 ± 1.544 and in the adequate group it was 9.021 ± 2.881. Comparing patients with deficient plasma vitamin C levels to those with adequate plasma vitamin C levels, the median PFS was 28 months for deficient group versus 23 months for the cancer-free group, respectively (*p* = 0.567) (Figure 1).

## 4. Discussion

This prospective cohort assessed the potential of plasma vitamin C at diagnosis to predict survival and progression among individuals with CRC after comparing levels to cancer-free controls. Results revealed that plasma levels were in line with those reported in the literature for other populations and CRC was an independent predictor of low plasma vitamin C. Moreover, even though progression and mortality tended to be higher in the deficient vitamin C group, these results did not reach statistical significance.

Mean plasma vitamin C levels were found to be 4.2 mg/l in the cancer group. Plasma vitamin C levels in participants with CRC are in accordance with those reported in the literature which ranged between 1.8 and 4.8 mg/l for cancers of the gastrointestinal tract (25–27). Mean plasma vitamin C levels in the cancer-free group were found to be 9.3 mg/l, in agreement with results reported from other trials which ranged between 6.8 mg/l and 12.1 mg/l (28–30). Comparing plasma vitamin C levels between cancer free participants and those with mCRC revealed that the cancer group had significantly lower plasma vitamin C levels. Moreover, the presence of cancer was found to be an independent predictor of diminished plasma vitamin C after adjusting for clinically relevant variables including age, gender, and vitamin C intake. In a case–control study nested within the European Prospective Investigation into Cancer and Nutrition (EPIC) study, Leenders et al. reported a significant difference in vitamin C levels between controls and participants with colon cancer (*p* = 0.03) similar to our study but found no significant difference between controls and individuals with rectal cancer which was different from our results (31). Moreover, Saygili et al. also reported that vitamin C levels of healthy subjects were double those of their peers with CRC (32). In our study, there was significant differences in vitamin C levels between colon and rectal sites compared to control when studied separately (data not shown). Moreover, dietary analysis revealed an intake of 172 mg/l in the cancer group compared to 144 mg/l in the control group, a difference which was not statistically significant. At the multivariate analysis, the presence of cancer was the only significant predictor of diminished vitamin C level with the cancer group having 5.4 times increased risk compared to cancer-free group, even after adjusting for vitamin C and caloric intake. Comparably, Leenders et al. reported a similar mean dietary intake of vitamin C of 106 mg/l in the CRC group compared to 109 mg/l among healthy controls (31). These results indicate that the lower plasma vitamin C levels seen among the participants with CRC is not related to the amount of dietary vitamin C consumed, but rather to the presence of CRC.

In our study, BRAF mutations occurred in 2 patients while RAS mutations (NRAS/KRAS) occurred in 24 patients. A previous study has found that mutant KRAS or BRAF CRC cells exhibit high expression of GLUT1, leading to increased uptake of the oxidized form of vitamin C, dehydroascorbate (DHA) (13). This increased DHA uptake is associated with increased oxidative stress as intracellular DHA is reduced to vitamin C, depleting glutathione and leading to an energetic crisis and cell death (13). However, our analysis revealed no significant difference in vitamin C levels in patients with BRAF mutations versus patients with no BRAF mutations and in patients with RAS mutations versus patients with no RAS mutations.

Our results showed a non-significant trend towards higher mortality in the vitamin C deficient CRC subgroup. Compared to oral administration of vitamin C, intravenous administration eludes the limited intestinal absorption, renal absorption and excretion. Padayatty et al. revealed that intravenous doses resulted in concentrations 100-fold higher than oral intake (33). To further explore the efficacy of vitamin C intravenous supplementation in individuals with CRC receiving cytotoxic therapy, a recent phase I study determined the maximum tolerated dose of vitamin C with mFOLFOX6 and FOLFIRI regimens in patients with metastatic colorectal and gastric cancer (34). The trial revealed that intravenous vitamin C administration at a dose of 1.5 g/kg once daily for 3 consecutive days in individuals receiving mFOLFOX6 or FOLFIRI with or without bevacizumab every 14 days exhibited a favorable safety profile in patients with mCRC (34). The efficacy of vitamin C is currently being explored alongside mFOLFOX  ±  bevacizumab as first-line therapy for patients with mCRC in a large phase III trial (35).

This prospective study had several strengths and limitations. On the one hand, it assessed plasma vitamin C level among patients and controls from the same population, determined vitamin C intake using a reliable FFQ specific to the Lebanese population, and assessed other clinically relevant variables such as physical activity level, comorbidities, performance score, etc. On the other hand, this cohort was limited by factors such as the absence of matching based on age and gender, and this was evident with the control group having a younger mean age and a higher percentage of females than the cancer group. This study was conducted amidst the COVID-19 pandemic. Hence, the choice of controls was limited to the hospital staff and family members accompanying patients to the hospital. However, these variables (age and gender) were adjusted for in the logistic regression, at the univariate and multivariate levels. This mismatch must be avoided at the design phase in future studies. Moreover, the small study sample may have affected the significance of the results since there was a trend for a higher mortality in the vitamin C deficient subgroup. The sample size was limited in this study by the availability of funds to assess vitamin C level. *Post hoc* power analysis revealed that 73 participants per arm were needed in the diminished and normal plasma vitamin C levels to detect statistically significant differences in mortality among participants with mCRC.

## 5. Conclusion

Our cohort study identified stage IV colorectal cancer as an independent risk factor for low plasma vitamin C levels compared to healthy controls from the same population after adjustment for several clinically relevant variables including age, gender and dietary intake of vitamin C and calories. Trends towards increased mortality and more prevalent BRAF and KRAS mutations were noted in the deficient vitamin C patients’ group. These differences did not reach statistical significance, possibly due to the small sample size. Future studies should validate findings in larger sample sizes and assess the effect of plasma vitamin C supplementation on survival and response to treatment in phase III clinical trials.

## Data availability statement

The raw data supporting the conclusions of this article will be made available by the authors, without undue reservation.

## Ethics statement

The studies involving human participants were reviewed and approved by The Institutional Review Board at the American University of Beirut. The patients/participants provided their written informed consent to participate in this study.

## Author contributions

ST: conceptualization, writing–original draft preparation, and supervision. JJ: writing–reviewing and editing, resources, and supervision. FN: investigation. RE and RH: data curation and formal analysis. MM: investigation. AE: data curation. MC: formal analysis and methodology. RN: resources and project administration. AS: conceptualization and supervision. All authors contributed to the article and approved the submitted version.

## Conflict of interest

The authors declare that the research was conducted in the absence of any commercial or financial relationships that could be construed as a potential conflict of interest.

## Publisher’s note

All claims expressed in this article are solely those of the authors and do not necessarily represent those of their affiliated organizations, or those of the publisher, the editors and the reviewers. Any product that may be evaluated in this article, or claim that may be made by its manufacturer, is not guaranteed or endorsed by the publisher.
